# Whole-genome sequencing reveals activation-induced cytidine deaminase signatures during indolent chronic lymphocytic leukaemia evolution

**DOI:** 10.1038/ncomms9866

**Published:** 2015-12-07

**Authors:** S. Kasar, J. Kim, R. Improgo, G. Tiao, P. Polak, N. Haradhvala, M. S. Lawrence, A. Kiezun, S. M. Fernandes, S. Bahl, C. Sougnez, S. Gabriel, E. S. Lander, H. T. Kim, G. Getz, J. R. Brown

**Affiliations:** 1 Department of Medical Oncology, Dana-Farber Cancer Institute, Brigham and Women's Hospital, Harvard Medical School, Boston, Massachusetts 02215, USA; 2 Department of Medicine, Brigham and Women's Hospital, Harvard Medical School, Boston, Massachusetts 02215, USA; 3 Cancer Program, Broad Institute of Harvard and MIT, Cambridge, Massachusetts 02142, USA; 4 Department of Biostatistics and Computational Biology, Dana-Farber Cancer Institute, Boston, Massachusetts 02215, USA; 5 Department of Pathology and Cancer Center, Massachusetts General Hospital, Boston, Massachusetts 02114, USA; 6 Department of Pathology, Harvard Medical School, Boston, Massachusetts 02215, USA

## Abstract

Patients with chromosome 13q deletion or normal cytogenetics represent the majority of chronic lymphocytic leukaemia (CLL) cases, yet have relatively few driver mutations. To better understand their genomic landscape, here we perform whole-genome sequencing on a cohort of patients enriched with these cytogenetic characteristics. Mutations in known CLL drivers are seen in only 33% of this cohort, and associated with normal cytogenetics and unmutated *IGHV*. The most commonly mutated gene in our cohort, *IGLL5*, shows a mutational pattern suggestive of activation-induced cytidine deaminase (AID) activity. Unsupervised analysis of mutational signatures demonstrates the activities of canonical AID (c-AID), leading to clustered mutations near active transcriptional start sites; non-canonical AID (nc-AID), leading to genome-wide non-clustered mutations, and an ageing signature responsible for most mutations. Using mutation clonality to infer time of onset, we find that while ageing and c-AID activities are ongoing, nc-AID-associated mutations likely occur earlier in tumour evolution.

## Abstract (web summary)

The oncogenic events driving indolent chronic lymphocytic leukaemia are relatively unknown. Here, the authors perform whole genome sequencing on 30 such tumours and identify recurrent mutations in *IGLL5* and two activation induced cytidine deaminase signatures that are operative at different stages of CLL evolution.

## Graphical abstract

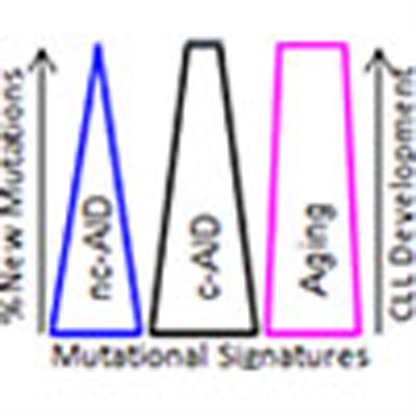

Chronic lymphocytic leukaemia (CLL) is a clinically heterogeneous incurable malignancy of CD5+CD19+B lymphocytes^1^. Among the strongest predictors of outcome are the disease-associated chromosome abnormalities, with 17p and 11q deletion and trisomy 12 associated with more aggressive disease, while 13q deletion (incidence 50–60%) and normal cytogenetics (incidence 15–20%) are lower risk according to Dohner's cytogenetic classification^2^. Interestingly, the recurrent coding mutations identified to date in CLL have been associated with the higher-risk cytogenetic abnormalities, and are less commonly seen in CLLs with a lower-risk cytogenetic profile. We therefore set out to explore the genetic basis of the lower-risk cytogenetic group by whole-genome sequencing, as clues to the genetic basis of disease in this more indolent group may lie elsewhere in the genome.

Whole-genome sequencing provides unique information not available from prior studies with whole-exome sequencing, including data on translocations, complex rearrangements and genome-wide mutational patterns. However, relatively higher sequencing costs have limited the number of whole-genome studies (*n*=4, Puente *et al.*^3^; *n*=28, Alexandrov *et al.*^4^, with only signature analysis reported without detailed cohort description) and to date, most studies involved larger exome data sets, which were likely the major driver of the primary findings. Here we present a comprehensive analysis of structural rearrangements and somatic mutations in 30 CLL whole genomes having low-risk cytogenetic aberrations. We deliberately balanced our cohort to evenly represent higher- and lower-risk *IGHV* cases, since different driver events might be relevant to these subgroups, as in fact turned out to be the case.

Recently developed techniques using Non-negative Matrix Factorization (NMF)^5^ to perform unsupervised analysis of somatic mutation data has enabled the unbiased discovery of genome-wide mutational patterns in multiple tumour types^4^,^6^,^7^. One such study, by Alexandrov *et al.*, analysed 28 CLL WGS and 103 whole-exome sequencing samples and found that CLL mutations comprise three mutational signatures: (i) ageing-related mutations (C>T at CpG mutations due to spontaneous deamination^4^; signature 1B); (ii) APOBEC signature (signature 2); and (iii) an activation-induced cytidine deaminase (AID)-related signature (signature 9). During B-cell development, AID induces deamination of cytosine to uracil. Resolution of these lesions by the error-prone DNA polymerase η (eta) results in A to C mutations at WA (W=A or T) motifs; described as signature 9 by Alexandrov *et al.*^4^ However, as noted by Alexandrov *et al.*^4^, signature 9 does not exhibit the known mutation features of canonical AID (c-AID) (C to T/G at WRCY motifs, W=A or T, R=purine, Y=pyrimidine)^8^, and is therefore referred to as a non-canonical AID (nc-AID) signature throughout this paper. Note that previously the c-AID signature was not separated as a distinct one in CLL^4^. However, prior experimental evidence has suggested that somatic hypermutation could be ongoing in a limited number of CLLs^9^. In addition, supervised mutation analysis^8^,^10^,^11^ did identify c-AID mutations in the immunoglobulin heavy chain locus in CLL^8^,^9^, as well as in multiple myeloma^10^ and diffuse large B-cell lymphoma (DLBCL)^11^. To the best of our knowledge, at the time of manuscript submission, genome-wide unsupervised discovery of c-AID signatures had not been performed in CLL.

In this study, our data set of 30 whole genomes provides the opportunity to perform an unsupervised analysis of the mutational patterns giving rise to indolent CLL. Here we present a modified Bayesian NMF algorithm that we have developed to analyse the mutation spectrum of CLL and show that it can successfully delineate both canonical and nc-AID signatures in an unsupervised genome-wide manner. In the context of known CLL and AID biology, our results support a model of differential activities of the two AID signatures and the ageing signature throughout tumour evolution.

## Results

### Structural rearrangements in CLL reveal chromoplexy events

**Figure 1:**
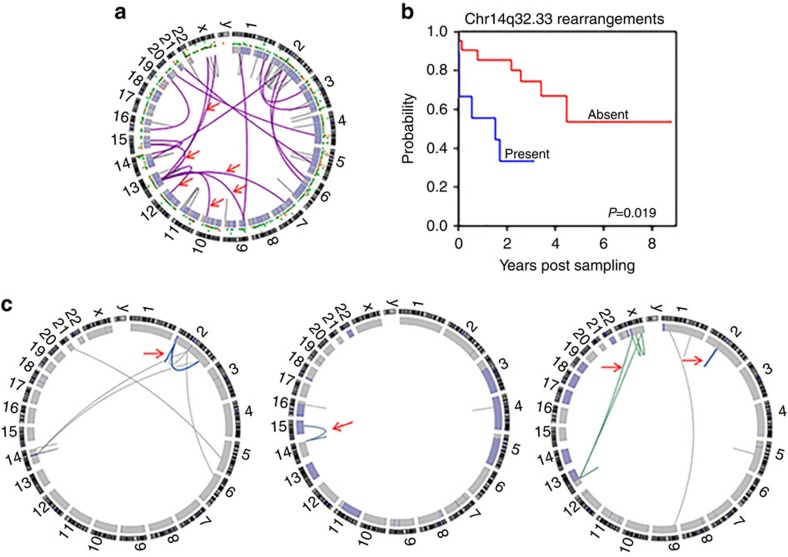
Summary of structural rearrangements. (**a**) Circos plot representing the structural rearrangements observed across 30 CLL genomes. Purple lines indicate inter-chromosomal rearrangements, grey lines indicate intra-chromosomal rearrangements; red arrows point to inter-chromosomal rearrangements giving rise to 13q deletion. (**b**) Kaplan–Meier curve showing the relationship between time to next treatment post sampling and rearrangements in Chr14q32.33 (5′IGH) in the vicinity of *KIAA0125*. (**c**) Circos plots depicting the presence of chained rearrangements detected by the ChainFinder algorithm. Red arrows indicate deletion bridges and inter-dependent chains. Left—1 chain near *LPIN1, TRIB2* and *TMEM194B* genes on chr2; middle–1 chain near *KIAA0125* on chr14 and *ANP32A* on chr15; right—chain 1 (blue) near *SNAR-H, REG3G and CTNNA2* on chr2 and chain 2 (green) near *ARMCX6, SAGE1, ZCCHC5* and *ITM2A* on chr23 and *CYSLTR2, EBPL, RNASEH2B* and *KPNA3* on chr13. The genes listed here either fall within a deletion or are within 25kb of a chained rearrangement breakpoint.

To assess the degree of genomic structural instability in the 30 CLLs genomes (see Supplementary Tables 1 and 2 for patient characteristics), we first analysed rearrangements, and identified a total of 92 events using the dRanger^12^ algorithm followed by BreakPointer^12^. This result corresponds to a median of 2.5 (range 0–15) rearrangements per genome, significantly fewer than most other cancers (Fig. 1a), underscoring the relative stability of these CLL genomes. Interestingly, deletion of 13q often occurred by an inter-chromosomal unbalanced translocation (6/16, 37.5% of 13q deletion cases, Fig. 1a) rather than a simple deletion (see Supplementary Data 1 for rearrangement partners). Hruba *et al.*^13^ reported a similar frequency of inter-chromosomal 13q rearrangements by fluorescence *in situ* hybridization. Apart from chr13, three other chromosomes were rearranged in ≥20% cases; chr2 (12 cases, 40%), chr14 (9 cases, 30%) and chr1 (6 cases, 20%). Chr14 accounted for 14% of rearrangements (13/92), mostly representing deletions. Eleven of these 13 rearrangements (9 cases) had break points at the 5′*IgH* region (chr14q32.33). Patients with chr14q32.33 rearrangements had a shorter time to next treatment post sampling (TTNT) (*P*=0.019, log-rank test, Fig. 1b). Although deletions in this locus have been frequently reported in a variety of B-cell neoplasms including CLL, they have not been detected in normal B cells, indicating that they are not a by-product of normal immunoglobulin rearrangements^14^,^15^. Single-nucleotide polymorphism (SNP) array analysis of the 30 CLLs revealed a median of 1 somatic copy-number alteration (sCNA) per case (range 0–6), similar to our previous report^16^. Other than 13q loss, we detected previously described sCNAs such as focal amplifications in 3q25.33 (ref. 15) in two cases (which include *PIK3CA)* and a focal deletion at 1q42.2 that was reported by Pfeifer *et al.*^17^

Next, we looked for complex structural rearrangements such as chromothripsis^18^ and chromoplexy^19^ since these may have disrupted multiple genes in a single event. While chromothripsis typically involves multiple focal deletions in a single chromosome (thought to occur during metaphase)^20^,^21^, chromoplexy is defined as a series of inter-dependent rearrangements among multiple chromosomes (most likely during interphase)^19^. Although we did not find evidence of chromothripsis, three of the 30 cases had evidence for chromoplexy (detected by ChainFinder^19^—an algorithm that links close rearrangements to balanced chains of events). Two of the cases had a single chain (both with three rearrangements) and one had two chains (with three and eight rearrangements) (Fig. 1c; Supplementary Data 2). In one case each, the chain included known common CLL copy-number changes, namely 13q deletion and 14q32 deletion. Interestingly, all three of these patients were untreated before sampling but underwent therapy shortly thereafter, suggesting that these events may indicate poor outcome (*P*=0.02, log-rank test), although this finding needs to be confirmed in a larger cohort. Our results indicate, for the first time, that chromoplexy events occur in CLL (involving 17 of the total 92 rearrangements). Earlier cytogenetic reports of chained translocations may also have reflected this phenomenon, albeit at much lower resolution. Statistical analysis of the copy-number and structural rearrangement data by the ChainFinder algorithm suggests that these events likely occurred at the same time and hence adds additional information beyond the previous cytogenetic studies. Taken together, our data suggest that in this indolent cohort a subset of 13q deletions may occur by inter-chromosomal rearrangement or even more involved chromoplexy events. Future larger studies are needed to correlate such events more definitively with clinical outcome.

### Increased subclonal mutation rate with age

**Figure 2:**
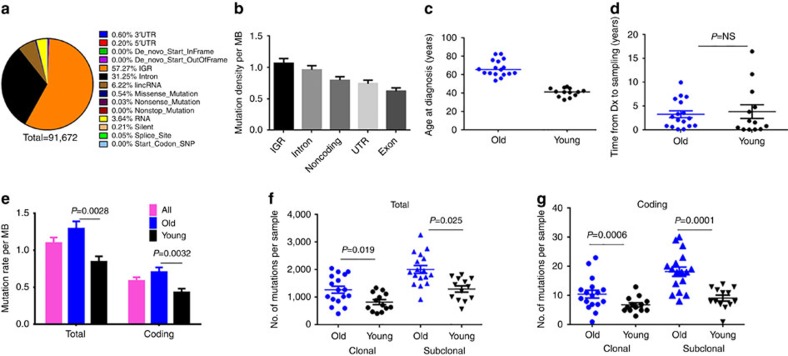
Overview of somatic mutational landscape. (**a**) Pie chart depicting the percentage of different types of sSNVs detected in our cohort genome-wide. (**b**) Bar chart of average mutation densities across different regions of the genome. *n*=30, error bars indicate ±s.e.m. (**c**) Dot plot of age at diagnosis in the older versus younger cohort. The horizontal line indicates median age. (**d**) Dot plot of time from diagnosis to sampling in the older versus younger cohort. (**e**) Bar chart comparing the mutation rate per MB genome-wide (total) and in coding regions in the entire cohort and in younger (*n*=13) versus older (*n*=17) patients. (**f**,**g**) Dot plot of average number of clonal and subclonal mutations total (**f**) and in coding regions (**g**) in younger versus older subgroups is shown. Error bars indicate ±s.e.m., *P* values were calculated using the Mann–Whitney *U*-test. NS, not significant (i.e. *P*>0.05).

Turning our attention to mutational patterns, we identified an average of 3,055 mutations per genome (Fig. 2a; Methods). The average genome-wide mutation frequency of 1.1±0.4 per Mb (range 0.4–2.1, *n*=30, data are shown as mean±s.d.) is lower than that of many other haematological malignancies and solid tumours^7^,^21^,^22^. The pattern of mutation densities (intergenic>intronic>untranslated region (UTR)>exonic) in different genomic regions was similar to other WGS studies^23^ (Fig. 2b).

Although CLL is largely considered to be a geriatric malignancy, about one-third of patients develop the disease much earlier. We have previously reported that disease diagnosed at an older age is associated with a higher number of clonal mutations (in coding regions^24^), but not subclonal mutations. Clonal mutations are likely to have occurred during life before onset of malignancy, while subclonal mutations arise subsequent to transformation, after the last selective sweep, and are therefore in only a subset of cells^24^. In this cohort, we deliberately selected older and younger patients matched for other disease characteristics, so as to better associate mutational patterns with age of diagnosis. We confirmed the expected increase in clonal mutations with age, but we were also able to observe a clearly higher number of subclonal mutations with older age (Fig. 2c–g), even though the disease duration was comparable in the young and old cohorts (Fig. 2d). Thus, in addition to the well-described higher clonal mutation burden acquired before disease development in older patients, we also find a higher ongoing rate of subclonal mutations, which may reflect more clonal evolution and heterogeneity.

### Somatic mutational landscape of indolent CLL by WGS

**Figure 3:**
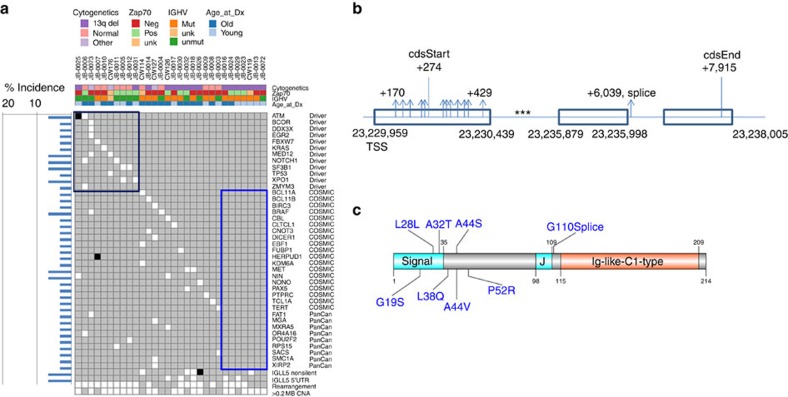
Distribution of mutations in selected genes. (**a**) Heatmap showing the presence of non-synonymous mutations in genes specified on the right. In the heatmap, white box = one event, black box=two events and gray=no events. The genes are classified based on potential functional significance as shown in the rightmost column. The top panel shows the clinical characteristics of each sample. The bar chart on the left indicates the percentage of cases with at least one mutation in the gene on the right. The bottom four rows in the heatmap represent the presence of mutations in IGLL5, rearrangement events and copy number alterations. The black box highlights samples with mutations in known CLL driver genes; the blue box highlights cases with no mutations in known cancer-associated genes. (**b**) Graphical representation of 5′UTR and coding mutations in the IGLL5 transcript. ***indicates mutations concentrated in the first intron. (**c**) Graphical representation of *IGLL5*-coding mutation alterations at the protein level.

Focusing on the specific somatic mutations, we observe that only 10 (33%) out of 30 patients displayed at least one mutation in a previously reported CLL driver. In comparison, 57% (91/160) of the cohort in our previous whole-exome study^24^ had at least one mutation in a CLL driver, indicating that this cohort does indeed capture a different biology (Fisher's exact test, *P*=0.027). Those patients with at least one driver mutation in a previously reported CLL cancer gene were more likely to have unmutated *IGHV* (*P*=0.014) and normal cytogenetics as compared with 13q deletion (*P*=0.033, Fisher's exact test). These patients also had a higher risk of progressing to next treatment (Hazard Ratio, HR=5.71, *P*=0.0076), as expected by their *IGHV* status. Interestingly, 7/30 cases (23%) did not harbour mutations in any gene previously associated with cancer (CLL drivers^24^, COSMIC or PanCancer^25^ mutations) (Fig. 3a); these patients all carried 13q deletion (Fisher's exact test, *P*=0.009) and five of them had mutated *IGHV.* The number of nonsilent mutations per tumour in these seven cases was also significantly lower than the rest of the cohort (13±5.5 versus 20±8.6, *P*=0.0027, Mann–Whitney *U*-test, data are shown as mean±s.d.). No difference in the number of rearrangements and sCNAs was seen in these two groups.

Interestingly, the 13q-deleted subgroup was enriched in 5′UTR and coding-region mutations in *IGLL5* (*P*=0.04, Fisher's exact test), the gene carrying the most frequent coding-region mutations in our cohort (Fig. 3b,c). These mutations were also more common in *IGHV*-mutated cases (*P*=0.013). Little is known about the function of the *IGLL5* gene, but it is homologous to *IGLL1* (lambda5), which is critical for B-cell development. Furthermore, *IGLL5* has been reported to be recurrently mutated in diffuse large B-cell lymphoma^26^. The mutation pattern in *IGLL5* was suggestive of off-target AID activity, with clustering of mutations near the transcription start site (TSS) through the first intron, as well as biallelic mutations (Supplementary Fig. 1a). The mutations included non-synonymous coding-region mutations (*n*=4), 5′UTR mutations (*n*=4) and one patient with both (total 9/30=30%), as well as 15 samples with mutations in the first intron (total 15/30=50%). The 5′UTR and coding mutations in *IGLL5* were enriched in subclonal mutations, whereas the intronic mutations were mostly clonal (*P*=0.006, Fisher's exact test), suggesting that the 5′UTR/coding mutations were acquired later than the intronic mutations, after the last selective sweep. The presence of 5′UTR and first exon mutations was confirmed by Sanger sequencing (*n*=7/8 cases, we did not have additional DNA from the 8th patient after sequencing). In addition, expression of the mutant alleles in the *IGLL5* coding region was also confirmed using matched RNA sequencing (RNA-seq) data (Supplementary Fig. 1b shows a representative Integrative Genomics Viewer [IGV] screenshot). *IGLL5* mutants showed a trend towards reduced transcript levels as compared with wild type (Supplementary Fig. 1c). Comparing the fraction of reads supporting the mutated allele in the WGS and RNA-seq data showed higher mutation allele fraction in the RNA-seq data in the coding mutations (*P*=0.0078), whereas the 5′UTR mutations had similar allele fractions (*P*=0.16) (Supplementary Fig. 1d). These data suggest a potentially different functional role for coding and 5′UTR mutations in *IGLL5*, but future experiments will be required to determine their true role if any in CLL pathogenesis. Given that *IGLL5* was the most commonly mutated gene in our cohort and the mutational pattern suggested the potential involvement of AID activity, we were interested in exploring more broadly the mutagenic processes, including AID, that give rise to the somatic mutations that lead to CLL^27^,^28^.

### Unsupervised discovery of mutational signatures

Normal B cells undergo somatic hypermutation in the germinal centre^29^—a process that is mediated by AID and induces clustered mutations in immunoglobulin loci and some off-target regions^10^,^30^. Following AID-induced deamination of cytosine to uracil, different repair processes lead to different mutational signatures, called either c-AID or nc-AID. Specifically, direct replication over the AID-induced U:G lesions or removal of the uracil by UNG (uracil DNA glycosylase) followed by replication accounts for the mutations of the c-AID signature (C to T/G mutation at WRCY motifs, W=A or T, R=purine, Y=pyrimidine; reviewed in ref. 31). Alternatively, processing of the AID-induced lesions by the mismatch repair pathway that recruits the error-prone DNA polymerase η gives rise to the nc-AID-related mutations (A to C/G at WA motifs^28^,^32^; reviewed in refs 31, 33).

**Figure 4:**
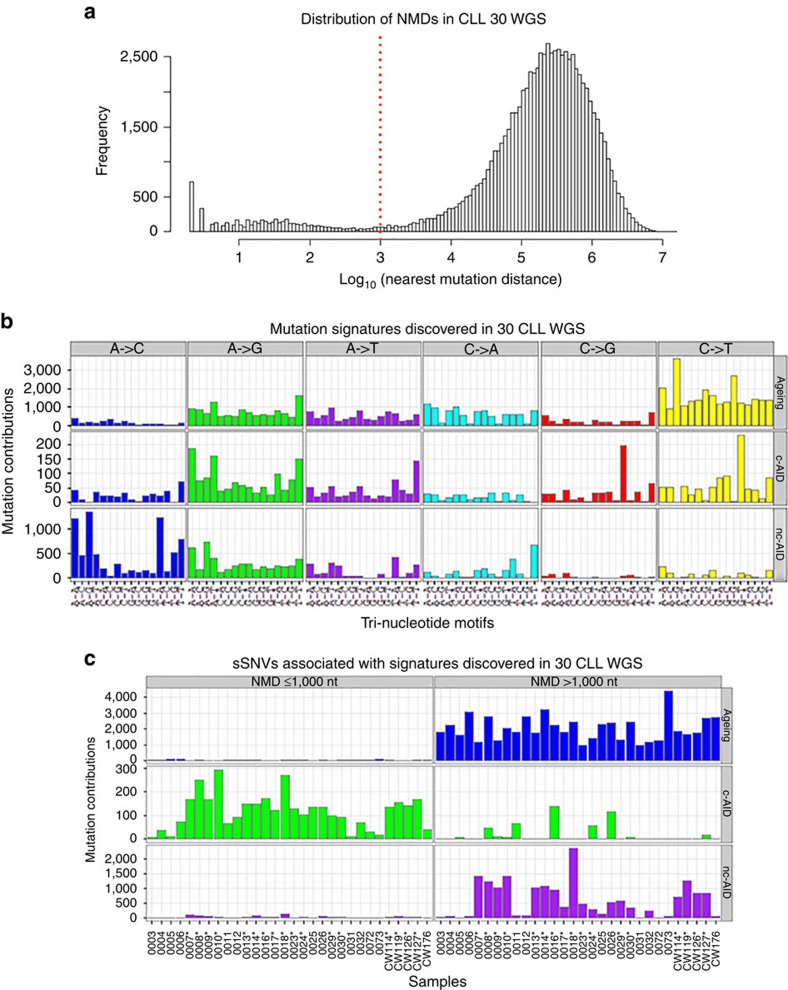
Analysis of mutational signature in CLL. (**a**) Frequency histogram of nearest mutation distance (NMD) shows bimodal distribution. (**b**) Estimated mutation contributions of the indicated mutational signatures detected upon inclusion of NMD as a factor in Bayesian NMF. (**c**) Number of clustered mutations (left) and non-clustered mutations (right) associated with canonical AID (green), ageing (blue) and non-canonical AID (purple) signature across samples. * Indicates cases with mutated *IGHV*.

Given our findings with *IGLL5*, and due to the known clustered nature of c-AID mutations^34^, we considered the nearest mutation distance (NMD—see Methods for details) as a parameter to stratify somatic mutations. We observed a bimodal distribution of mutation distance that enabled a partitioning of the mutations into two groups: (i) a clustered group (NMD<1,000 nt) consisting of 7% of mutations and (ii) a non-clustered group (NMD>1,000 nt) with the remaining 93% of mutations (Fig. 4a). Comparing the mutational spectra of these two groups revealed a marked increase of C>T/G at GCT motifs in the group of clustered mutations. This pattern of mutations matches the known c-AID signature^7^, suggesting that this process contributes to the mutational load in CLL (Supplementary Fig. 2). Although AID expression has been reported in only 0.01–2% of quiescent circulating CLL cells^35^, our finding is consistent with previous studies that have reported AID activity in CLL cells by analysing intra-clonal *IGHV* diversity^36^,^37^ and induction of *de novo* somatic hypermutation *in vitro*^8^,^38^.

**Figure 5:**
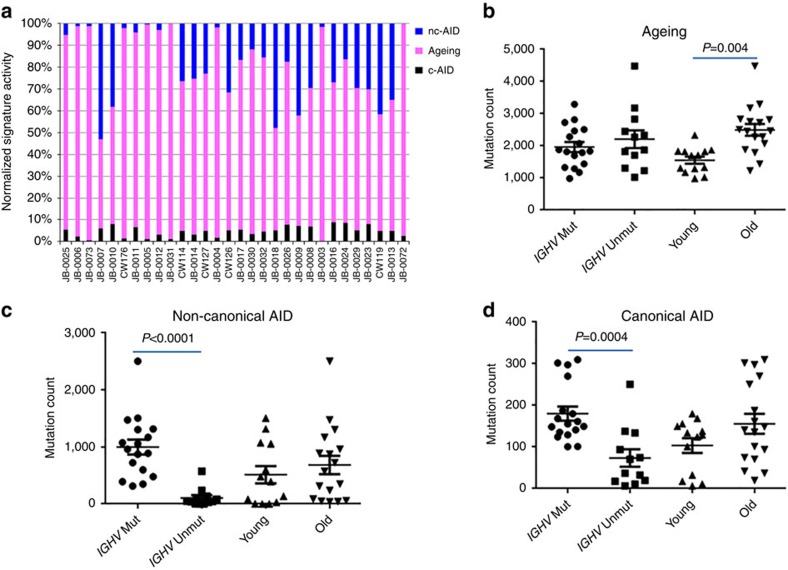
Association of signatures with clinical characteristics. (**a**) Percentage contribution of each of the mutational signatures to the overall mutation spectrum across samples. (**b**–**d**) Dot plots showing total mutation counts associated with the indicated signatures in relation to age at diagnosis (younger versus older) and *IGHV* mutation status (mut versus unmut). Error bars indicate±s.e.m. P values were calculated using the Wilcoxon's Rank Sum Test.

Recently, Alexandrov *et al.*^4^ identified, in an unsupervised manner, 21 mutational signatures across 30 different tumour types by applying NMF to the mutation counts across the 96 available trinucleotide mutation contexts. Here we characterized the mutational signatures operating in our 30 CLL cases using a related Bayesian NMF method^5^ considering NMD as an additional feature. Thus, instead of analysing a 96-by-30 matrix of mutation counts, we partitioned the mutations in each tumour into two groups of clustered and non-clustered mutations, giving rise to a 96-by-60 matrix (Methods). This partitioning enabled the discovery of mutational signatures unique to the clustered and non-clustered mutations. Our analysis identified three mutational processes, only two of which were reported by Alexandrov *et al.*^4^: an ageing signature characterized by increased C>T transitions at CpG sites (analogous to their signature 1B); a nc-AID signature, dominated by A>C at WA motifs (analogous to their signature 9) (Figs 4b,c and 5a); and a third signature that matches the c-AID signature (C to T/G mutation at WRCY motifs, W=A or T, R=purine (A or G), Y=pyrimidine (C or T)) that was not reported by Alexandrov *et al.*^4^ To further validate the finding of the c-AID signature, we reanalysed the 28 WGS CLL samples from Alexandrov *et al.* using our method and were able to validate both the c-AID and nc-AID signatures in their data (Supplementary Fig. 3), although the c-AID signal was not as strong as in our cohort. Thus, our analysis provides definitive evidence that c-AID activity in CLL is strong enough to be discovered in an unsupervised analysis of genome-wide mutational patterns.

Next, we calculated for each mutation, m, the probability (*p*_ms_) that it was generated by each of the three mutational signatures, s, and assigned it to a signature if that probability (*p*_ms_) was greater than 0.75 (Methods). As expected, plotting the NMD along the genome for each signature revealed that the c-AID-associated mutations form distinct clusters, whereas the nc-AID- and ageing-associated mutations are scattered more evenly (Supplementary Fig. 4). From this analysis, we were able to determine that the three signatures exhibit differential contribution to the overall mutational landscape of each patient. The ageing signature was predominant across all cases and the number of ageing-related mutations was significantly higher in patients with older age at diagnosis, as might be expected (Fig. 5b, *P*=0.004, Wilcoxon's rank-sum test). However, 70% of cases had at least 10% of mutations due to AID activities (Fig. 5a). The number of mutations due to c-AID and nc-AID was significantly higher in *IGHV*-mutated CLLs (Fig. 4c and Fig. 5c,d; *c-AID P*=0.0004, *nc-AID P*<0.0001, Wilcoxon's rank-sum test). Consistent with this, 7/12 *IGHV*-unmutated cases showed >95% ageing signature. Among coding mutations, 95% were associated with the ageing signature (*p*_m,ageing_>0.75), whereas only 1.9 and 2.4% were associated with c-AID and nc-AID, respectively. Therefore, the ageing signature is likely to be the primary contributor to driver mutations in coding regions in CLL. Interestingly, the seven samples with no mutations in known CLL drivers or other cancer genes showed a lower number of ageing-associated mutations (*P*=0.021, Mann–Whitney test).

### c-AID signature exhibits classical features of SHM

**Figure 6:**
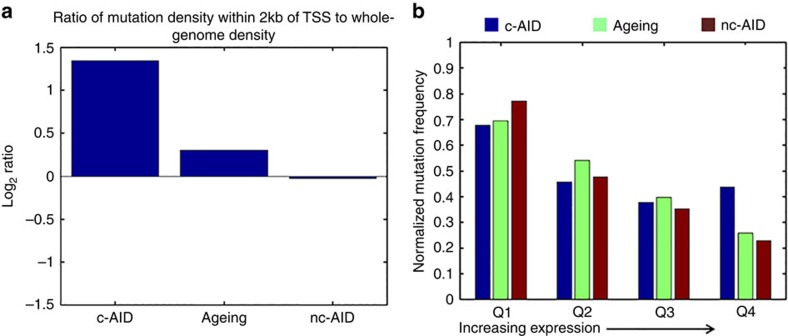
c-AID mutations exhibit classical features of SHM. (**a**) Ratio of mutation frequency within 2 kb of transcription start site (TSS) to the genome-wide mutation rate for each signature. (**b**) Genes were divided into four quartiles, Q1 through Q4, in order of increasing expression. Bar graph showing normalized mutation density of each signature per quartile.

Apart from the WRCY recognition motif, other previously described characteristics of off-target c-AID activity include (1) multiple and biallelic mutations (evident from the observed clustering and the mutation pattern in c-AID targets such as *IGLL5*) and (2) preferential targeting ±2 kb from the TSS of highly transcribed genes (reviewed in ref. 31). We observe that the c-AID-associated mutation rate was increased 2.5-fold within 2 kb of TSS as compared with the genome-wide rate (Fig. 6a). To confirm the preference for highly transcribed genes, we divided the genes into four quartiles based on their expression levels determined by RNA-seq, and compared the contribution of the three signatures with the mutation rate in genes in each quartile. As expected, the overall rate of mutations decreases with higher expression levels due to transcription-coupled repair. However, the c-AID mutation rate was found to be the highest compared with the other two signatures in the genes in the quartile with the highest expression (Q4) (Fig. 6b).

### Identification of genome-wide targets of the AID signatures

Next, we focused on the contribution of c-AID and nc-AID to the mutational density in individual genes (including UTRs and introns), to find specific target genes unique to each of the AID processes. Specifically, we identified non-overlapping sets of trinucleotide sequence contexts that distinguish the c-AID and nc-AID signatures (Supplementary Fig. 5a) (using only mutations with *p*_ms_>0.75). For each AID signature, we then compared the observed mutation density in every gene with at least one signature-associated mutation (*p*_ms_>0.75, 281 c-AID and 809 nc-AID genes) to the context-specific background mutation density in these genes (Methods, Supplementary Fig. 5). We then corrected for multiple hypotheses and identified genes associated with each signature using a *q*-value cutoff of 0.1 (Supplementary Fig. 5b).

For c-AID, we detected 34 associated genes with *q*<0.1 (Supplementary Data 3). Consistent with known AID biology, 24 (70%) of these genes were located in the cytobands with the three immunoglobulin loci (14q32.33, 22q11.22 and 2p11.2). Unsurprisingly, *IGLL5* was one of the most significant genes associated with the c-AID signature in this analysis (*q*<10^−90^, Supplementary Data 3). The list also included *BCL6* and *LTB*, other known off-targets of AID in post-germinal centre B-cell malignancies^39^,^40^ and another haematologic malignancy-related gene, *TRIP11* (ref. 41) (Supplementary Data 3).

For nc-AID, we discovered 14 genes that were specifically targeted (*q*<0.1, Supplementary Data 4). This list includes two genes in the immunoglobulin cytobands as well as cancer-related genes such as *CADM2* (renal cell carcinoma)^42^, *CHRM3* (colon cancer)^43^, *LPHN3* (panCancer analysis)^44^ and *ROBO1* (breast cancer)^45^ (Supplementary Data 4). The biologic basis of this signature selectivity and its relevance to cancer development will need to be clarified in future studies.

### Ageing and c-AID activities are ongoing in CLL

**Figure 7:**
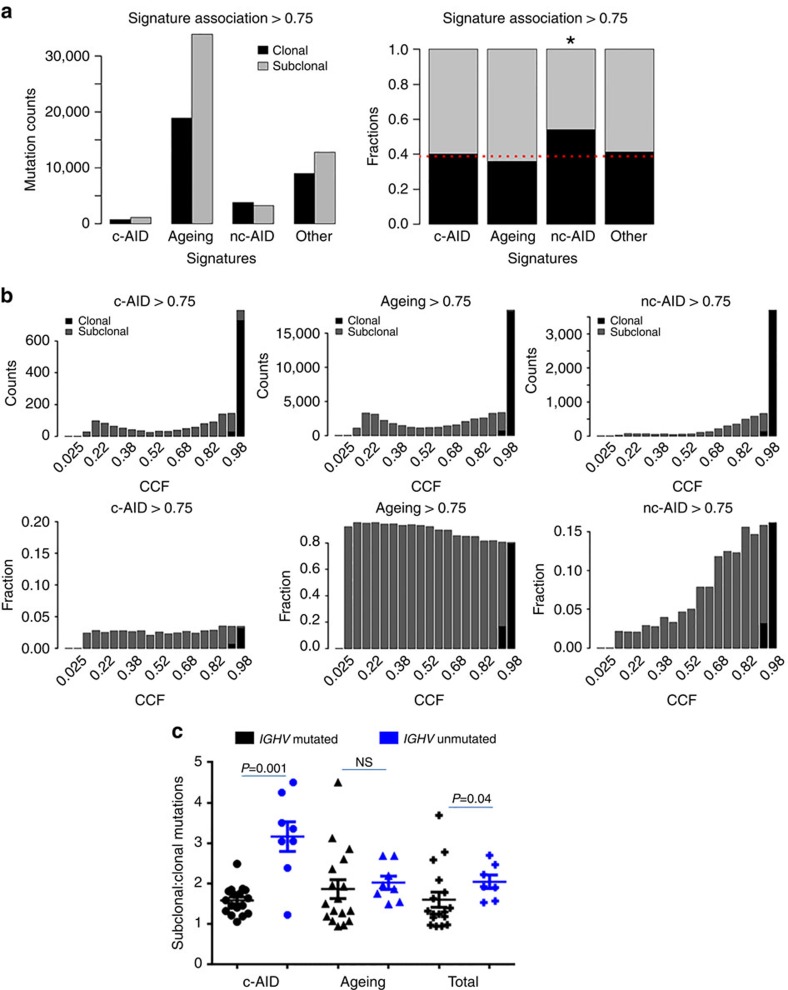
Chronological order of mutational processes. (**a**) Bar graph showing absolute number (left) and ratio (right) of clonal and subclonal mutations in the indicated categories (*p*_ms_>0.75). ‘Other' includes mutations that were not assigned to any of the three signatures. *P<0.000001, P value was calculated using the Chi-square Test. (**b**) Distribution of CCF of mutations assigned to each signature; total number (top) and the fraction (bottom) of mutations for given CCF. (**c**) Ratio of subclonal:clonal mutations among mutations associated with either c-AID or ageing, compared with total mutations, shown divided by *IGHV* status. Note that *p*_ms_>0.5 is shown here (*P*=0.001) since *p*_ms_>0.75 had a low overall *n*, but a similar trend was observed with *p*_ms_>0.75, *P*=0.055. We only considered cases with at least five c-AID-associated mutations, resulting in *N*_(*IGHV* mut)_=17 and *N*_(*IGHV* unmut)_=8 for *p*_ms_>0.5. NS, not significant. (i.e. P<0.05). Error bars indicate±s.e.m. P values were calculated using the Mann Whitney *U* Test.

We analysed mutation clonality to assess the activity of these three mutational processes over time during the life history of the CLLs. The clonality of a given mutation can be used to deduce the time of its onset in relation to the most recent selective sweep, with clonal mutations being earlier events and subclonal mutations occurring later. We used ABSOLUTE^46^ to assess clonal versus subclonal status (Methods) and examined the proportion of clonal and subclonal mutations associated with each mutational process (*p*_ms_>0.75) (Fig. 7a). Overall, we found a significant association between clonality and the mutational processes (*P*<0.00001, *χ*^2^-test). Next, we tested each mutational process independently and found that each signature had a different proportion of clonal mutations. The nc-AID signature had the highest proportion of clonal mutations (*P*<0.00001, Fisher's exact test). On the other hand, c-AID-associated mutations were equally distributed between clonal and subclonal populations (*P*=0.26, Fisher's exact test), and ageing was enriched in subclonal mutations (*P*<0.00001, Fisher's exact test).

Given this difference in the proportion of clonal mutations in each signature, we were interested in using these data to infer the time of onset of each of the mutational processes in the life history of the CLL. To do this, we looked at the distribution of the proportion of tumour cells bearing a signature-associated mutation, namely, the cancer cell fraction (CCF) for each mutation. We plotted the fraction of mutations associated with each signature as a function of their CCF. This analysis showed that 54% of the nc-AID mutations were clonal (high CCF). As expected, therefore, the fraction of nc-AID-associated mutations declined sharply at low CCF values, indicating relatively few subclonal mutations, and suggesting that nc-AID was more active at earlier stages of tumour evolution (Fig. 7b; Supplementary Fig. 6). In contrast, roughly 40% of the c-AID mutations were clonal, and c-AID mutations showed a constant proportion across CCF values, suggesting both early and ongoing c-AID activity. Ageing-associated mutations were least likely to be clonal, with only 36% clonal, and, consistent with that, ageing-associated mutations showed a slight increase towards lower CCF, that is, more representation in subclonal mutations. These data therefore suggest that nc-AID occurred mostly at earlier times, whereas c-AID and ageing are continuing to operate even after the last selective sweep.

### Ongoing c-AID activity is enriched in unmutated *IGHV* cases

Being a strong mutator, AID activity is tightly regulated in cells^47^. It is expressed in a very small fraction of circulating CLL cells, likely those in the proliferative fraction. Interestingly, and unexpectedly given that more somatic hypermutation is present in mutated *IGHV* CLLs, this expression of AID (encoded by the *AICDA* gene) among circulating CLL cells has been shown to be enriched among unmutated *IGHV* cases^48^,^49^. In our present cohort, although AID mRNA expression is very low overall, its expression is significantly higher in unmutated *IGHV* (Supplementary Fig. 7, *P*=0.001, Mann–Whitney *U*-test). Hence, we hypothesized that the ongoing c-AID activity evident in the low-CCF subclonal c-AID-associated mutations would be enriched in *IGHV*-unmutated patients. In fact, the ratio of c-AID-associated subclonal:clonal mutations was higher in the unmutated compared with mutated *IGHV* CLLs (Fig. 7c, *p*_ms_>0.5, *P*=0.001; *p*_ms_>0.75, *P*=0.055, Mann–Whitney *U*-test, Supplementary Data 5 and 6), even though the overall frequency of c-AID-associated mutations was higher in mutated *IGHV* CLLs (Fig. 5d). These data suggest that the ongoing c-AID activity is enriched in unmutated *IGHV* CLLs, even though the sum total of c-AID activity across all of tumour evolution is enriched in mutated *IGHV* CLLs. As a control, we assessed whether the ratio of ageing-associated subclonal:clonal mutations was associated with *IGHV* status, and found no association (Fig. 7c).

## Discussion

In summary, we describe here the results of whole-genome sequencing of a CLL cohort comprised of low-risk cytogenetic subgroups in which we find that a small subset have complex rearrangements that may be associated with more aggressive disease, while a significant number have only 13q deletion as an obvious CLL driver.

Unlike previous studies^24^, we find significant enrichment in not just clonal but also subclonal mutations with age. Acquisition of subclonal mutations or clonal evolution has been associated with worsening disease^24^,^50^. While this supports the paradigm of acquisition of passenger mutations with age, it also points towards a more heterogeneous tumour in older patients and/or a faster ongoing acquisition of new mutations within the tumour. Thus, the age-associated increase in clonal diversification may be a key factor promoting worse disease outcomes in older patients.

We discovered recurrent mutations in *IGLL5* that were previously undescribed in CLL. Interestingly, these mutations segregate independently of the known CLL driver genes and thus seem to be a unique feature of low-risk CLL. The pattern of *IGLL5* mutation is suggestive of off-target AID activity, which is more prominent in lower-risk *IGHV*-mutated CLL. The mutations are expressed and were associated with a trend towards lower overall gene expression. Although the complete functional characterization of this protein is beyond the scope of this manuscript, we have presented several indicators that suggest *IGLL5* mutations may be of biological importance. Taken together, these findings point towards a potential functional role of *IGLL5* perturbation in low-risk CLL. However, further experimental work is required to confirm any such role.

Systematic analysis of mutational signatures gives insights into key mutagenic processes governing the developmental history of a cancer cell. Using a novel signature discovery method that uses information on both sequence context and mutation distance, we were able to identify three mutational signatures operative in CLL, including two distinct AID processes (c-AID and nc-AID) that represent a greater fraction of mutational activity in mutated *IGHV* cases, and the ageing-related signature. Somatic hypermutation is a critical physiological process in B-cell development responsible for affinity maturation of antibodies^51^. This process is initiated by AID, a 24-kDa protein that catalyses cytosine deamination to produce uracil, thereby creating U:G mismatches^52^. Repair of AID-induced lesions give rise to C to T/G mutations at WRCY motifs—termed as c-AID, and A to C/G mutations at WA motifs—termed as nc-AID^51^. Although AID activity is tightly regulated to primarily target the immunoglobulin-variable region genes, off-target AID activity can cause oncogenic mutations and chromosomal instability^51^. Despite scattered data suggesting that c-AID activity is present in CLL, the c-AID mutational signature was not identified in a previous unsupervised analysis of mutational signatures for CLL^4^. Here we demonstrate that the c-AID activity is separable as a distinct mutational signature using genome-wide unsupervised analysis considering the mutation distance as an additional feature.

Recently, Pettersen *et al.* reported c-AID-induced mutations in kataegis regions in CLL using a supervised motif discovery method^7^. A similar supervised motif discovery in four CLL whole genomes by Rebhandl *et al.* had previously implicated APOBEC activity in CLL^53^. Although APOBEC is widely expressed in CLL, our unsupervised signature discovery did not yield an APOBEC mutational footprint in our cohort. We applied the supervised motif analysis, as per Rebhandl *et al.*^53^, to our cohort, and were unable to detect evidence for APOBEC activity in clustered and non-clustered mutations at either immunoglobulin or non-immunoglobulin loci.

Analysis of whether a mutation is clonal or subclonal can be used to infer the time of occurrence of that mutation in relation to initial malignant transformation, with clonal mutations occurring earlier. We therefore examined the clonal fraction of mutations associated with each of our signatures. The ageing signature activity was enriched in patients with late-onset disease and enriched in subclonal mutations, which occur later, therefore suggesting that the ageing signature is a source of ongoing mutagenesis in CLL. This finding is consistent with our observation of not just increased clonal, but also increased subclonal mutations with age. Interestingly, the nc-AID-associated mutations were more clonal, suggesting that this process primarily occurred before the last selective sweep and perhaps even before cancer initiation. The mutation clonality analysis suggested that c-AID activity represents both an early and an ongoing process in the CLL life cycle. The higher proportion of newer subclonal c-AID-related mutations in *IGHV*-unmutated CLL, suggests that ongoing c-AID activity is higher in this subgroup. These findings are consistent with prior work showing that in mature circulating CLL cells, AID activity is more easily induced in unmutated *IGHV* patients, and hence more likely to create newer mutations^8^. It should be noted that the sum total of all AID-related mutations is significantly higher in the mutated *IGHV* cases, as also reported by Alexandrov *et al.*^4^

Circulating CLL cells are mostly in the G0/G1 phase^54^,^55^, although recent work has demonstrated that a small pool of proliferating cells is always present^56^, likely arising from tissue niches. Our data suggest that DNA-polymerase-η-mediated repair of AID-induced genetic lesions, which results in nc-AID mutations, occurs predominantly earlier in the CLL life cycle, perhaps even before transformation, while UNG-mediated repair, which results in c-AID mutations, is ongoing early and later. Little is known about the factors governing the relative activity and timing of pol-η and UNG in repairing AID-mediated strand breaks. DNA pol-η which contributes to nc-AID mutations, is more active in the S phase^57^,^58^. *UNG*, which contributes to c-AID mutations, is most abundant in the G1/S transition and S phase^59^, but it is possible that in somatic hypermutation and class-switch recombination, *UNG* exerts its function in G1, similar to AID^47^,^60^. Specifically, using a mouse model, Sharbeen *et al.*^61^ have shown that the mutagenic activity of *UNG* on AID-induced lesions was exclusively restricted to the G1 phase. These data may suggest that pol-η is more active in the S phase, while *UNG* is more active in G1; how this paradigm applies during the early development and transformation of a B cell to a CLL cell is yet unclear, but our data suggest that pol-η activity is earlier in this process.

In summary, by characterizing a lower-risk CLL cohort with WGS, we were able to show for the first time the operation of a distinct c-AID signature in CLL using unsupervised genome-wide analysis, and to demonstrate that this signature is in fact more abundant in cases with lower-risk mutated *IGHV*. These cases have fewer driver mutations and their key causative events beyond deletion 13q are still unclear. We are continuing to analyse noncoding and promoter regions from these whole genomes, as well as to correlate these results with epigenetic analyses, in an effort to identify the key driving events in these indolent CLL patients. Meanwhile, our new mutational signature detection method can be extended to other cancers to better elucidate signatures associated with clustered mutations.

## Methods

### Sample preparation

Matched peripheral blood (tumour) and saliva (normal) samples were collected after obtaining informed consent to a tissue banking protocol approved by the Institutional Review Board at Dana-Farber Cancer Institute (protocol no. 99–224). For samples with white blood cell count <25 K or absolute lymphocyte count <20 K, B cells were purified using the Easy Sep Human B cell Enrichment Kit (StemCell Technologies Inc., Vancouver, Canada) according to the manufacturer's instructions before viably freezing. Tumour and saliva DNA were extracted using QIAamp Blood DNA (Qiagen Inc., Valencia, CA) and Oragene DNA (Oragene, Ontario, Canada) kits, respectively, according to the manufacturer's directions.

### Whole-genome sequencing

Purified DNA was submitted to the Genomics Platform at the Broad Institute (Cambridge, MA) for high-throughput whole-genome sequencing. All samples were subjected to in-house quality control (QC) procedures such as Picogreen-based double-stranded DNA quantification (Life Technologies, Carlsbad, CA) and fingerprinting to confirm the match between a tumour and its intended normal, before library preparation.

For a subset of samples, starting with 3 μg of genomic DNA, library construction was performed as described by Fisher *et al.*^62^ Another subset of samples, however, was prepared using the protocol by Fisher *et al.*, with some slight modifications. Initial genomic DNA input into shearing was reduced from 3 μg to 100 ng in 50 μl of solution. In addition, for adapter ligation, Illumina paired-end adapters were replaced with palindromic forked adapters with unique eight-base index sequences embedded within the adapter. For a subset of samples, size selection was performed using gel electrophoresis, with a target insert size of either 340 or 370 bp±10%. Multiple gel cuts were taken for libraries that required high sequencing coverage. For another subset of samples, size selection was performed using Sage's Pippin Prep.

Following sample preparation, libraries were quantified using quantitative PCR (kit purchased from KAPA biosystems) with probes specific to the ends of the adapters. Cluster amplification was performed according to the manufacturer's protocol (Illumina) using either HiSeq 2000 v2, or HiSeq v3 cluster chemistry and flowcells. For a subset of samples, after cluster amplification, SYBR Green dye was added to all flowcell lanes, and a portion of each lane was visualized using a light microscope, to confirm target cluster density. Flowcells were sequenced on HiSeq 2000 using HiSeq 2000 v2 or v3 Sequencing-by-Synthesis kits, then analysed using RTA v1.10.15. or RTA v.1.12.4.2.

Mean target coverage of 30X and 60X was achieved for the tumour and normal samples, respectively. Pilot analysis of two normal saliva samples to determine the percentage of bacterial DNA contamination suggested that 60X coverage would be adequate to achieve 30X human DNA coverage in our samples. Average length of the paired-end reads was 101 bp with an 8-bp index. The raw sequence reads were processed and aligned to the hg19 human reference genome using the ‘Picard' pipeline, followed by QC using ‘Firehose' tools developed at the Broad^22^ (https://www.broadinstitute.org/cancer/cga/Firehose). The QC parameters tested include lane cross-check fingerprinting for sample identity, tumour normal cross-contamination measured using ContEst^63^ and coverage statistics. All samples passed the QC check.

### Identification of somatic mutations

High-confidence somatic mutation calls were made by applying MuTect^64^ to whole-genome sequencing data from tumours and patient-matched normal samples. Refer to Cibulskis *et al.*^64^ for more details. In addition, commonly occurring germline variants were filtered out using a panel of normals. The somatic mutation calls were further subjected to a realignment filter to remove remaining false-positive calls (Supplementary Data 7 for genome-wide somatic single nucleotide variants (sSNV) calls).

### Estimation of clonality using ABSOLUTE

Tumour samples are frequently contaminated with normal cells. ABSOLUTE^46^ infers the purity and ploidy of this heterogeneous population using copy-number and mutation data. ABSOLUTE also estimates local copy number in the cancer cells and the CCF of each mutation (that is, the fraction of cancer cells harbouring the mutation). We followed the same procedure as described in Landau *et al.*^24^ (Supplementary Data 8). Specifically, mutations with probability≥0.5 of having CCF≥0.95 were classified as clonal, and the rest were classified as subclonal. Mutations with CCF <0.1 were filtered out due to low power.

### Discovery of structural rearrangements

Clusters of discordant read pairs were used to infer the presence of structural rearrangements using the dRanger^11^ and BreakPointer^11^ algorithms. Mapped distance between pairs that is greater than that expected, based on library insert-size distribution, indicated the presence of a deletion. Inter-chromosomal rearrangements were identified as mate-pairs with each end mapping to different chromosomes. Tandem duplications were identified as pairs with same orientation, as well as an unexpected insert size. dRanger uses a panel of 177 whole-genome-sequenced normals to filter known germline rearrangements and artefacts. The algorithm assigns a final score based on number of supporting read pairs and a series of filtering matrices described in greater detail previously^11^. A score cutoff ≥4 was selected, as previous work has shown that it yields at least 85% true positives in a large-scale PCR-based validation study^11^. Breakpointer can be downloaded at https://www.broadinstitute.org/cancer/cga/breakpointer. See Supplementary Data 1 for a list of structural rearrangements.

### Analysis of SNP array data

A minimum of 250 ng of tumour and matched normal DNA was used to run Affymetrix Genome-Wide Human SNP Array 6.0 containing 906,600 SNPs and more than 946,000 probes for the detection of copy-number variation on a single genotyping array. The Genome-Wide Human SNP Array 6.0 uses a Birdsuite calling pipeline that delivers SNP as well as CNA calls. Germline CNAs and artefacts were removed by normalizing against a panel of normals. The resultant copy number segments file (seg file) with log2 copy-number ratios was used for further analysis. The number of sCNAs per sample was calculated manually using the following parameters, followed by visual inspection in IGV and comparison with germline: segment length ≥0.2 MB, amplification threshold ≥0.1, deletion threshold ≤−0.1.

The structural rearrangement data and SNP array data were modelled together using the ChainFinder^18^ algorithm to detect inter-dependent events, as described in detail by Baca *et al.*^18^ The algorithm is available at https://www.broadinstitute.org/cancer/cga/chainfinder. See Supplementary Data 2 for ChainFinder output.

### Discovering mutational signatures

The mutation signatures discovery is a de-convolution process of the somatic mutation counts in each tumour, stratified by mutation contexts and potentially other biologically meaningful parameters, into a set of characteristic mutational signatures. Here we applied the Bayesian NMF algorithm (BayesNMF)^5^ to infer the number of mutational signatures and their sample-specific contributions. In addition to raw mutation counts stratified by 96 base substitutions in trinucleotide sequence contexts, we also considered the clustering information of mutations as an additional feature in the signature discovery. We considered the NMDs, a minimum genomic distance to all other mutations on the same chromosome in the same patient, as a parameter to stratify mutations, and partitioned them into ‘clustered' (NMD≤1,000 nt) and ‘non-clustered' groups (NMD>1,000 nt) (Fig. 4a). The comparison of overall mutation spectrum between clustered and non-clustered mutation groups (Supplementary Fig. 4) revealed a significant elevation of C>T/G at GCT context and A>G at WA (W=A/T) in the clustered mutation group, corresponding to the known AID mutation motifs. On the basis of this observation, we separately counted clustered and non-clustered mutations across 96 trinucleotide mutation contexts in each sample. We split mutations in each tumour into two columns representing clustered and non-clustered mutational groups, giving rise to the mutation count matrix **X** (96 by 2*M*, *M*=the number of samples). This mutation count matrix was ingested as an input for the BayesNMF and factored into two matrices, **W′** (96 by K) and **H′** (K by 2M), approximating **X** by **W′H′**. It should be noted that clustered and non-clustered mutations from the same patient were separately handled to capture a characteristic signal from clustered mutations. While the conventional NMF requires the number of signatures *K a priori*, BayesNMF automatically prunes away irrelevant components that do not contribute to explaining **X** and effectively determines the appropriate number of *K*. We ran BayesNMF 50 times with exponential priors for **W′** and **H′** and 41 out of the 50 runs converged to the solution of *K*=3, while 9 runs converged to the solution of *K*=4. We used the three-signature solution (*K*=3) with the maximum posterior for the downstream analysis. To enumerate the number of mutations associated with each mutation signature, we performed a scaling transformation, **X∼W′H′**=**WH**, **W**=**W′U**^**−1**^ and **H=UH′**, where **U** is a *K*-by-*K* diagonal matrix with the element corresponding to the 1-norm of column vectors of **W′**, resulting in the final signature matrix **W** and the activity matrix **H**. Note that the *k*th column vector of **W (*****w***_***k***_) represents a normalized mutability of 96 trinucleotide mutation contexts in the *k*th signature and the *k*th row vector of **H (*****h***_***k***_) dictates the estimation of clustered and non-clustered mutations associated with the *k*th signature across samples (Fig. 4c).

### Signature-enrichment analysis

Using the determined **W** and **H** from the BayesNMF, we annotated each mutation with the probability (likelihood of association) that it was generated by each of the discovered mutational signatures, *p*_ms_, where ‘m' denoted a mutation and ‘s' refers to the signature. More specifically, the likelihood of association to the *k*th signature for a set of mutations corresponding to *i*th mutation context and *j*th clustered or non-clustered mutation group was defined as [***w***_***k***_***h***_***k***_/Σ_*k*_***w***_***k***_***h***_***k***_]_*ij*_, where ***w***_***k***_ and ***h***_***k***_ correspond to the *k*th column vector and *k*th row vector of **W** and **H**, respectively.

For the gene-level signature-enrichment analysis, we first attempted to identify a hotspot mutation motif out of 96 contexts in each signature by considering mutations only with *p*_ms_ >0.75. Note that keeping mutations with a higher *p*_ms_, filtered out mutations shared by multiple signatures and enabled the discovery of more distinct mutation motifs unique to each signature. By considering contributions more than the third quintile, we were able to extract characteristic mutation motifs to each signature—19 hotspot mutation motifs in c-AID and five hotspot mutation motifs in nc-AID (Supplementary Fig. 5a). To take into account sequence composition variation across the genome, we enumerated all available trinucleotide contexts across genes having non-zero mutations with *p*_ms_>0.75 in each signature. This information was used to estimate the background mutation rates at the hotspot motifs in each signature, resulting in *r*_cAID_=0.27 per Mb and *r*_ncAID_=0.58 per Mb for c-AID and nc-AID signatures, respectively. Then, for given mutation counts, *x*, at hotspot motifs and available sequence context, *n*, in each gene, we performed a binomial test with the estimated background mutation rate to assess the significance of the enrichment of each signature across 281 genes for c-AID and 809 genes for nc-AID having non-zero mutations with *p*_ms_ >0.75 (Supplementary Fig. 5b; Supplementary Data 3 and 4). We corrected for multiple hypotheses and identified genes that are associated with each signature using a *q*-value cutoff of 0.1 (see *Q*–*Q* plots in Supplementary Fig. 5c).

Two threshold values (0.5 and 0.75, Supplementary Data 5 and 6) for *p*_ms_ were utilized for the clonality analysis to dichotomize the signature association of mutations.

### RNA sequencing and analysis

RNA was extracted using the Qiagen RNeasy kit and the RNA integrity number was measured using Agilent Bioanalyzer at the Harvard BioPolymers Facility to assess the quality of the extracted RNA. Only samples with a RNA Integrity Number >8 were submitted for sequencing. Poly-A-selected RNA was used for library construction using the Illumina TruSeq Paired End Strand-specific kit according to the manufacturer's protocol and sequenced using Illumina HiSeq. The RNA-seq BAMs were aligned to the hg19 genome using TopHat^65^ (Gencode gtf used for annotation). QC analysis was performed using the metrics described by DeLuca *et al.*^66^ Gene-level expression data represented as fragments per kilobase of exons mapped was obtained using Cufflinks^67^.

### Statistical analysis

Statistical analysis was performed using with SAS version 9.2 (SAS Institute, Cary, NC) and R version 2.15.2 (the CRAN project). Categorical variables were compared using the Fisher's exact test or a *χ*^2^-test as appropriate, and continuous variables were compared using the Wilcoxon's rank-sum test. TTNT was defined as the time of sampling to the first treatment after sampling or death, whichever occurs first. Patients who did not receive a treatment after sampling were censored at the date last known alive and without any treatment. TTNT was estimated using the Kaplan and Meier method, and the difference was tested using the log-rank test. In addition, univariable Cox modelling was performed for known CLL risk factors as well as exploratory factors presented in this paper. Due to the limited number of events, multivariable Cox modelling was not explored. The linearity assumption for continuous variables was examined using restricted cubic spline estimates of the relationship between the continuous variable and log-relative hazard, and the cutoff points of these variables were based on the change of the log-relative hazards. All *P* values are two sided and considered significant at the 0.05 level. Due to the exploratory nature, multiple comparisons were not adjusted in the significance level.

## Additional information

**Accession codes:** The sequence data have been deposited in dbGAP under the accession code phs000879.v1.p1 http://www.ncbi.nlm.nih.gov/projects/gap/cgi-bin/study.cgi?study_id=phs000879.v1.p1.

**How to cite this article:** Kasar, S. *et al.* Whole-genome sequencing reveals activation-induced cytidine deaminase signatures during indolent chronic lymphocytic leukaemia evolution. *Nat. Commun.* 6:8866 doi: 10.1038/ncomms9866 (2015).

## Supplementary Material

Supplementary Information Supplementary Figures 1-7, Supplementary Tables 1-2 and Supplementary Reference

Supplementary Data 1 Structural rearrangements in CLL discovered using the dRanger algorithm

Supplementary Data 2 Inter-dependent rearrangements identified using the ChainFinder algorithm

Supplementary Data 3 Gene level association of c-AID

Supplementary Data 4 Gene level association of nc-AID

Supplementary Data 5 Number of mutations associated with each signature at pms>0.75

Supplementary Data 6 Number of mutations associated with each signature at pms>0.5

Supplementary Data 7 Genome wide somatic single nucleotide variants (sSSNV)

Supplementary Data 8 List of genome-wide clonal and subclonal somatic single nucleotide variants (sSSNV)
