# Supplementary material for: Whole-genome sequencing reveals activation-induced cytidine deaminase signatures during indolent chronic lymphocytic leukaemia evolution
Source: Nat Commun. 2015 Dec 7;6:8866. doi: 10.1038/ncomms9866 (PMC4686820; doi:10.1038/ncomms9866)
Supplement: Supplementary Information — Supplementary Figures 1-7, Supplementary Tables 1-2 and Supplementary Reference [file ncomms9866-s1.pdf]

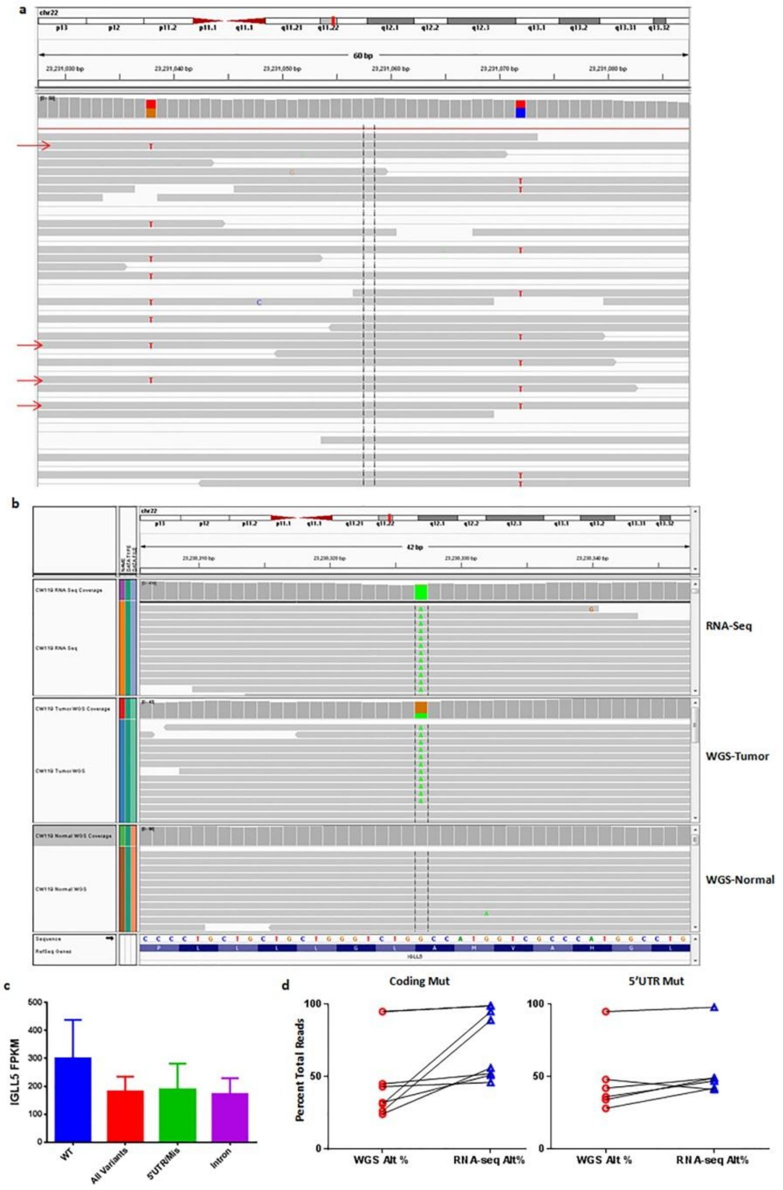

Supplementary Figure 1: Features of *IGLL5* Mutations in CLL: a) Representative IGV screenshot of first intron *IGLL5* mutation depicting biallelic mutations. Red arrows highlight the presence of out of phase mutations indicating hits in both alleles. b) Representative IGV screenshot of an *IGLL5* mutant. Tracks from top to bottom –RNA-seq, Tumor WGS and Normal WGS. c) Bar chart of IGLL5 mRNA expression levels measured using RNA-Seq. N_{WT} = 13, $N_{All\ Variants}$ = 17, $N_{5'UTR/Mis}$ = 9, N_{Intron} = 8. Error bars indicate ± s.e.m. d) Percentage of IGLL5 alternate alleles in WGS and RNA-seq data in coding (left) and 5'UTR (right) mutations.

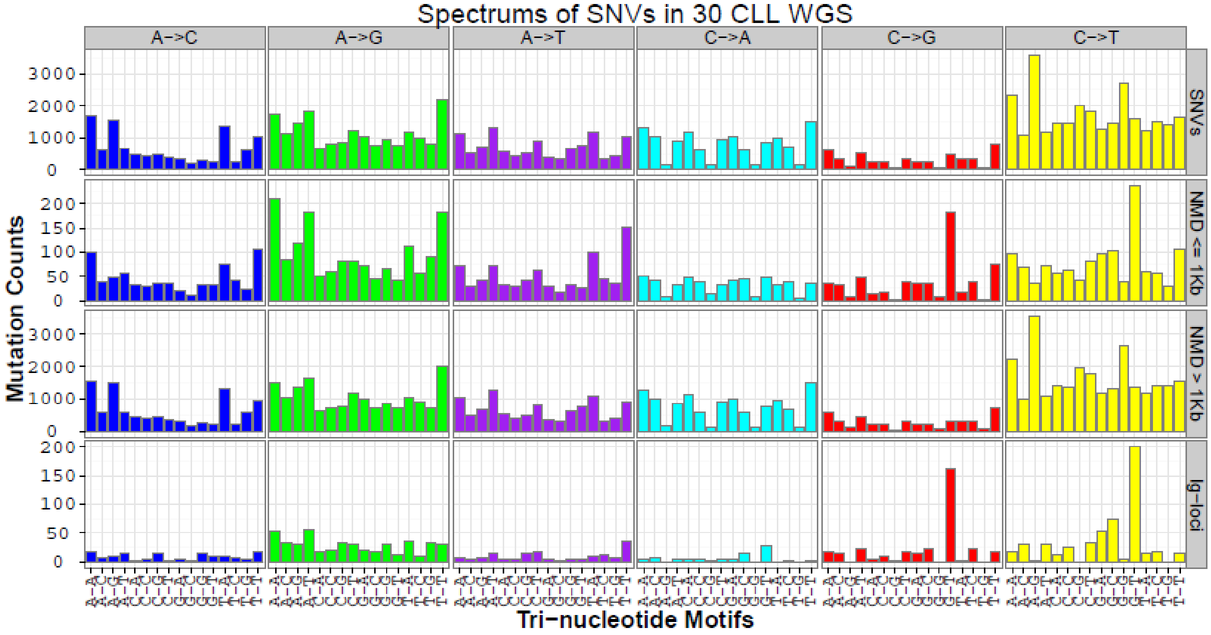

Supplementary Figure 2: Mutation Clustering across the Genome: Histogram of mutation counts across 96 base substitutions in tri-nucleotide context. Y-axis (top to bottom) represents the mutation counts in all SNVs, clustered SNVs (NMD<=1000nt), nonclustered SNVs (NMD>1000nt), and clustered mutations in three Ig loci.

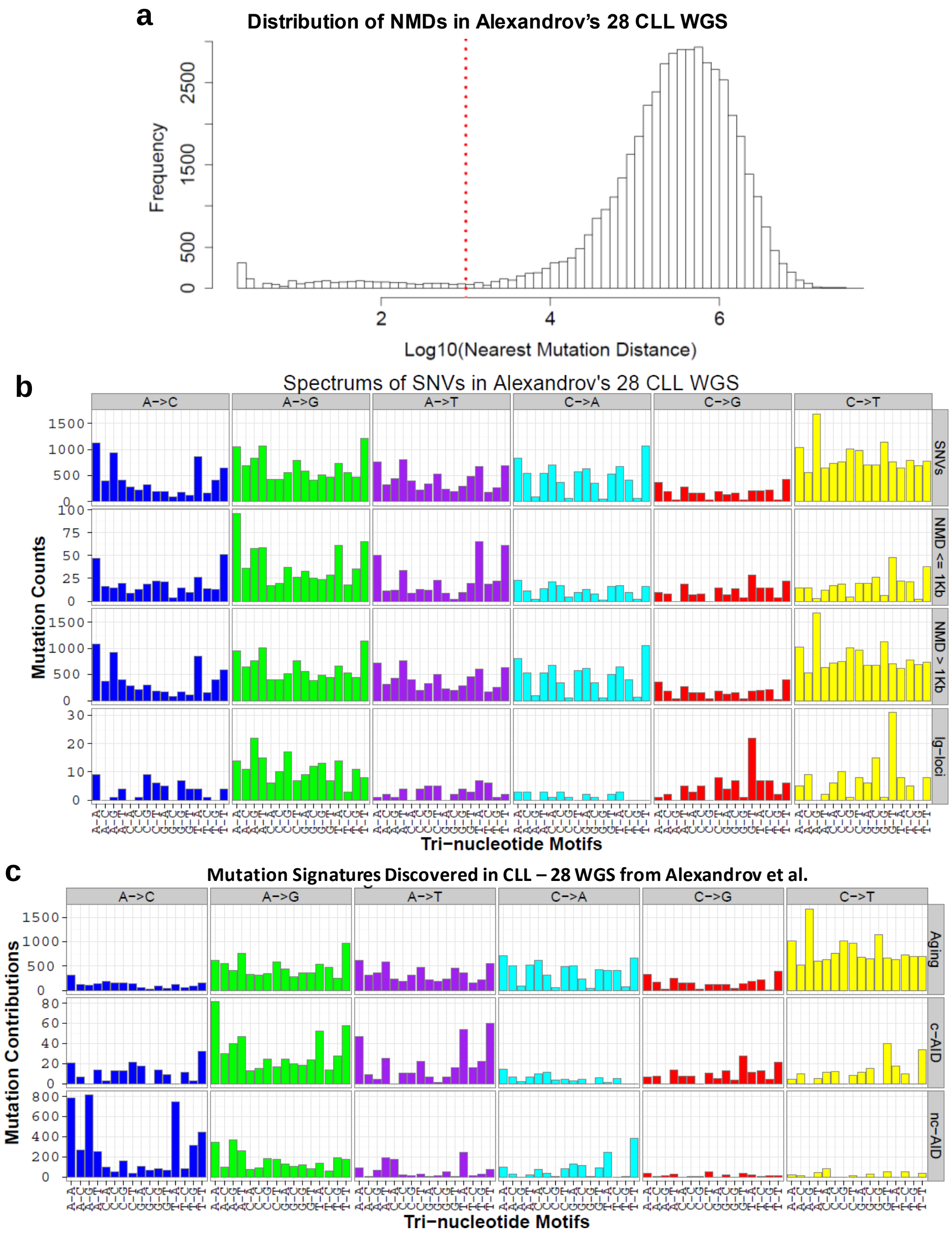

Supplementary Figure 3: Mutation Signature Discovery in Validation Cohort: a) Frequency histogram of nearest mutation distance (NMD) shows bimodal distribution in the dataset from Alexandrov et al[1]. b) Tri-nucleotide frequency of the human genome displayed using a 96 substitution matrix. Y-axis (top to bottom) represents the mutation counts in all SNVs, clustered SNVs (NMD<=1000nt), d) nonclustered SNVs (NMD>1000nt) and clustered mutations in Ig loci. c) Normalized contribution of the indicated mutational signatures detected upon inclusion of NMD as a factor in Bayesian NMF.

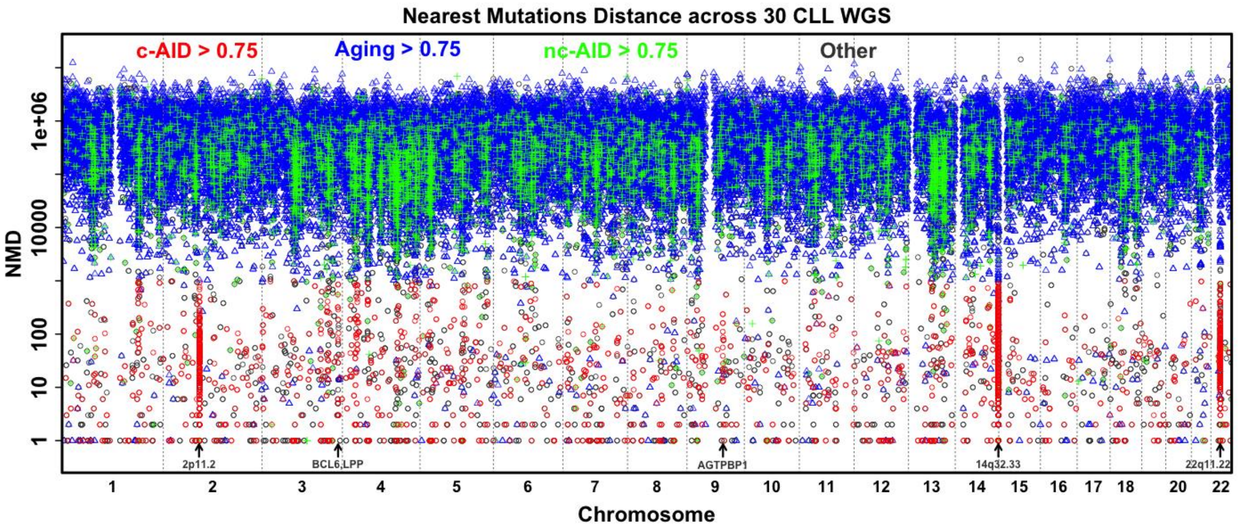

Supplementary Figure 4: Genome-wide Distribution of Signature Specific Mutations: Nearest mutation distance (NMD) of all sSNVs in 30 CLL cases is plotted according to its genomic co-ordinates (X-axis). Arrows point to specific clusters of mutations at the three immunoglobulin loci (2p11.2, 14q32.33 and 22q11.22) and at non-immunoglobulin loci (*BCL6*, *LPP* and *AGTBP1*).

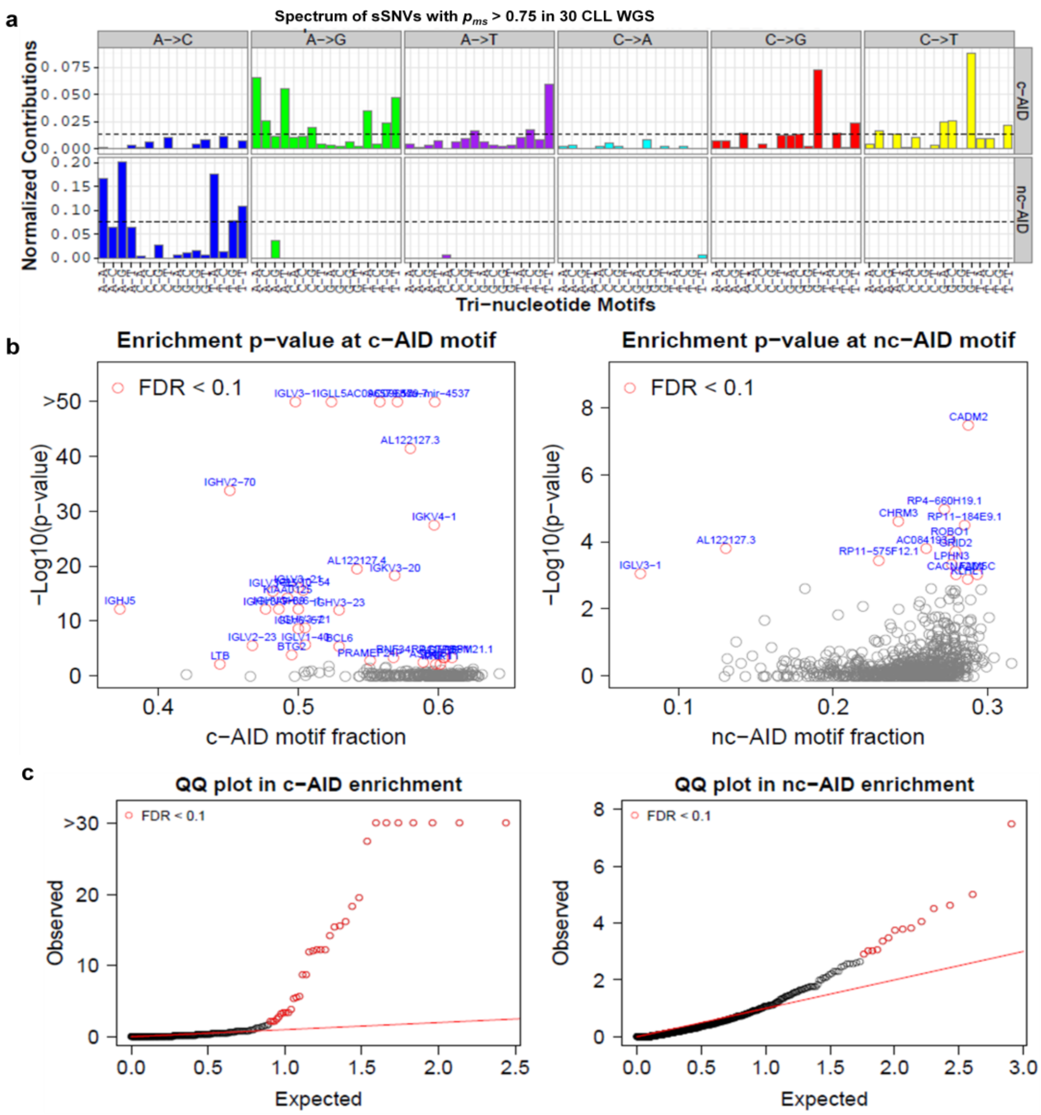

Supplementary Figure 5: Identification of c-AID and nc-AID Hotspot Genes: a) Normalized contribution of the indicated mutation spectrum observed upon considering only mutations with p_{ms}>0.75. The dashed horizontal lines indicated the third quantile in each normalized spectrum. This led to the discovery of non-overlapping tri-nucleotide contexts between the two AID processes. b) The background mutation rate for nc-AID and c-AID was calculated as described in the methods, followed by a binomial test to compare the observed and background mutation rate. The log p values thus obtained were plotted against the fraction of each motif. Red circles indicate hotspot genes (q<0.1). c) Q-Q plot of signature enrichment test per gene, X-axis is Expected -$\log_{10}$pvalue and Y-axis is Observed -$\log_{10}$pvalue.

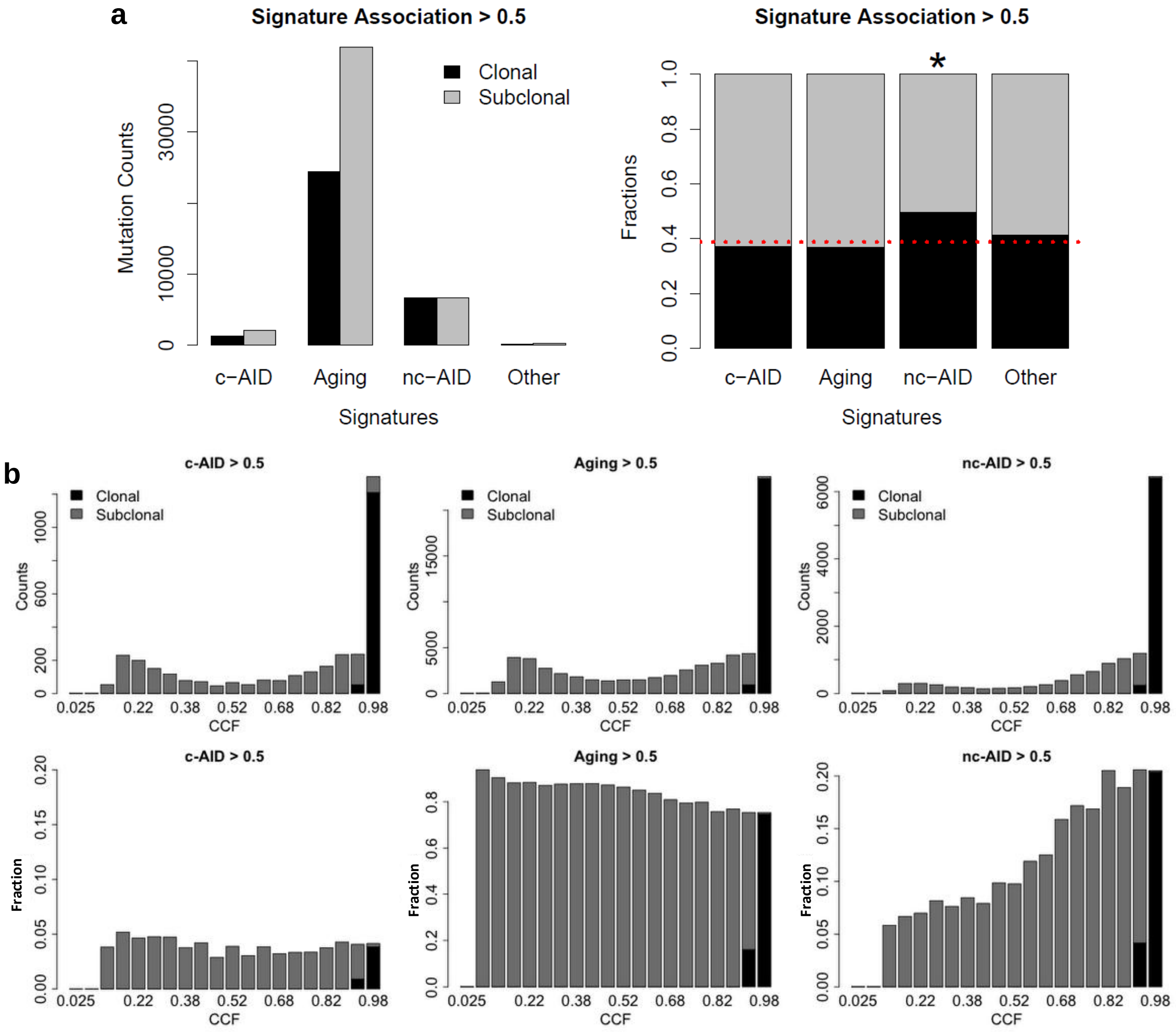

Supplementary Figure 6: Chronological Order of Mutational Processes at p_{ms}>0.5: a) Bar graph showing absolute number (left) and ratio (right) of clonal and subclonal mutations in the indicated categories (p_{ms}>0.5). b) Distribution of CCF of mutations assigned to each signature. Top panel, total number of mutations; bottom panel, proportion of mutations.

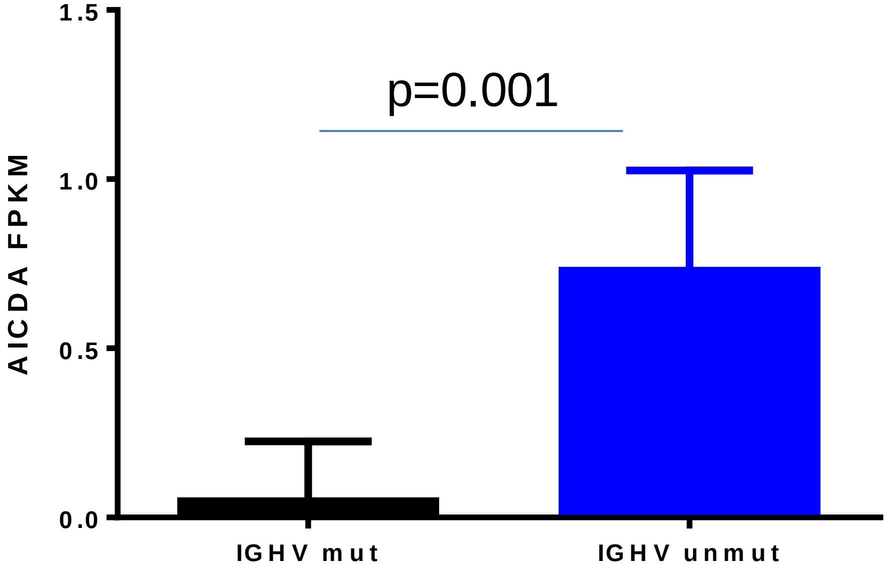

Supplementary Figure 7: Expression of AICDA in our Cohort: Bar chart depicting AICDA mRNA expression in mutated (N=17) versus unmutated IGHV (N=12) CLL. p=0.001; Mann Whitney U test; error bars indicate ±s.e.m.

	N	%
Total	30	100
Age at Dx, median (range)	58 yrs (33-83)	
Young (33-47 yrs)	13	43
Old (54-83 yrs)	17	57
IGHV Mutational Status		
Mut	17	57
Unmut	12	40
Unk	1	3
Zap70		
Negative	16	53
Positive	11	37
Unk	3	10
Cytogenetics		
Normal	11	37
13q(del)	16	53
Others	3	10
Median Time from Dx to Sampling (range)	2 yrs (0.09-16.5)	
Median follow-up from Dx (range)	5.8 yrs (1.2-20.5)	
No. of Treated Patients	13	43
Median Time from Dx to 1st Tx (range)	2.5 yrs (0.04-10.4)	

Supplementary Table 1: Summary of Patient Characteristics: Others in cytogenetics represents 1 case each of 17p del, 11q del and Trisomy12; Dx = Diagnosis; Tx = Treatment.

Sample	Age at Dx	Young=0, Old=1	*IGHV* (unmut = 0, mut =1)	ZAP (Neg = 0, Pos = 1)	Cytogenetics (Normal=0, 13q=1, Other=2)
CW114	61	1	1	unk	0
CW119	47	0	1	unk	1
CW126	62	1	1	1	1
CW127	62	1	1	0	2
CW176	60	1	unk	unk	2
JB-0003	41	0	0	0	0
JB-0004	62	1	0	0	0
JB-0005	45	0	0	1	0
JB-0006	82	1	0	1	0
JB-0007	34	0	1	0	0
JB-0008	83	1	1	0	0
JB-0009	41	0	1	0	0
JB-0010	54	1	1	0	0
JB-0011	42	0	0	1	0
JB-0012	68	1	0	1	0
JB-0013	33	0	1	0	1
JB-0014	77	1	1	0	1
JB-0016	74	1	1	0	1
JB-0017	36	0	1	0	1
JB-0018	67	1	1	0	1
JB-0023	45	0	1	1	1
JB-0024	61	1	1	1	1
JB-0025	42	0	0	0	1
JB-0026	56	1	0	0	1
JB-0029	46	0	1	1	1
JB-0030	76	1	1	1	1
JB-0031	38	0	0	1	1
JB-0032	66	1	0	1	1
JB-0072	41	0	0	0	1
JB-0073	65	1	0	0	2

Supplementary Table 2: Clinical Characteristics per Case

Supplementary Reference

1. Alexandrov, L.B. *et al.* Signatures of mutational processes in human cancer. *Nature* **500**, 415-21 (2013).
